# Aldehyde Detoxification in Cancer: Metabolic Guardians of the Genome

**DOI:** 10.3390/biom16070991

**Published:** 2026-07-06

**Authors:** Brandon T. James, Emma P. Kosmeder, Meng Wang

**Affiliations:** 1 Division of Nutritional Sciences, Cornell University, Ithaca, NY 14853, USA; btj33@cornell.edu (B.T.J.);; 2 Biomedical and Translational Sciences, College of Veterinary Medicine, Cornell University, Ithaca, NY 14853, USA

**Keywords:** aldehyde detoxification, aldehyde dehydrogenases, metabolic genotoxins, genome instability, DNA repair, cancer

## Abstract

Reactive aldehydes are potent electrophiles that damage DNA and can ultimately promote cancer. While many aldehydes are ubiquitous in the environment, mammalian metabolism also generates high levels of endogenous aldehydes sufficient to threaten genome integrity. To protect against these aldehyde genotoxins, mammalian cells rely on diverse families of aldehyde detoxification enzymes as well as DNA repair pathways. Here, we review how aldehyde detoxification safeguards genome integrity, focusing on the aldehyde dehydrogenase (ALDH) family, alcohol dehydrogenase 5 (ADH5), and the glyoxalases. We discuss how these enzymes operate in a 2-tier model (in which tier 1 detoxification enzymes cooperate with tier 2 DNA repair pathways in a tissue-specific manner), the consequences for cancer initiation and progression when aldehyde detoxification is compromised, and the opportunities for targeting aldehydes in cancer therapy.

## 1. Introduction

Genome instability is a fundamental hallmark of cancer [1], underpinning the accumulation of mutations that drive malignant transformation and progression. For decades, research into genome instability in cancer has focused on defects in DNA damage sensing, signaling and repair/tolerance pathways [2]. Loss-of-function mutations in genes such as breast cancer gene 1 and 2 (*BRCA1/2*), the mismatch repair genes underlying Lynch syndrome, and the Fanconi anemia pathway (FA pathway) genes are well-established drivers of cancer predisposition through their failure to maintain genome integrity [3]. However, this repair-centric view addresses only one side of the equation: how cells respond to DNA damage once it has occurred. Comparatively less attention has been paid to the upstream question of what generates the genotoxic damage in the first place, particularly from endogenous sources.

It is now clear that normal cellular metabolism produces a range of reactive species capable of damaging DNA. The reactive oxygen species (ROS) generated during mitochondrial respiration represent perhaps the best-studied example [4]. Superoxide dismutase (SOD), catalase, and the glutathione peroxidase system constitute a well-characterized enzymatic defense against oxidative DNA damage [5], and the consequences of their dysfunction, including the accumulation of 8-oxo-deoxyguanosine lesions and increased mutagenesis, are firmly established [6]. Reactive aldehydes are another class of endogenous genotoxins [7]. Aldehydes are potent electrophiles capable of forming covalent adducts with DNA bases, DNA-protein crosslinks (DPCs), and DNA interstrand crosslinks (ICLs). Environmental exposure to aldehydes, including formaldehyde (an industrial chemical and pollutant) and acetaldehyde (from metabolism of ingested alcohol), has been associated with increased cancer risk since the 1980s, and both are now classified as Group 1 human carcinogens by International Agency for Research on Cancer (IARC) [8]. Aldehydes are also produced endogenously through core metabolic processes, including one-carbon metabolism [9,10], glycolysis [11], and lipid peroxidation [12]. In response to these diverse DNA lesions, mammalian cells rely on multiple DNA repair pathways to protect their genomes against aldehyde-induced DNA damage [13,14]. Upstream from DNA repair, genotoxic aldehyde-DNA lesions are prevented from forming through aldehyde clearance by detoxification enzymes. The importance of these enzymes in maintaining genome integrity and suppressing cancer development is now becoming more apparent.

Mammals have evolved a diverse family of aldehyde detoxification enzymes. Aldehyde dehydrogenase (ALDH) enzymes directly catabolize free aldehydes [15,16], whereas alcohol dehydrogenase 5 (ADH5) [17,18] and glyoxalases (glyoxalase 1 (GLO1) and hydroxyacylglutathione hydrolase (HAGH)) [19] process aldehyde–glutathione conjugates. Many of these enzymes exhibit overlapping substrate specificity, adding complexity to deciphering which aldehydes are the relevant physiological substrates in vivo. Nonetheless, the discovery that genetic deletion or loss-of-function of these enzymes in mammalian cells leads to increased DNA damage has been pivotal in establishing aldehyde detoxification as a parallel axis of genome protection alongside DNA repair [20,21,22]. In both mouse models and humans, the genomic instability arising from the loss of aldehyde detoxification is sufficient to drive increased cancer development, thus highlighting both the carcinogenic potential of endogenous aldehydes and the critical need for aldehyde detoxification in limiting malignant transformation [23,24]. Beyond cancer initiation, large-scale transcriptomic analyses of established human cancers have revealed that aldehyde detoxification genes are frequently dysregulated, with expression changes that correlate with patient survival, indicating roles for aldehyde detoxification in cancer progression and response to treatment. Here, our review focuses on aldehyde detoxification enzymes (ALDHs, ADH5, and glyoxalases) as guardians of mammalian genome integrity. We examine the evidence that these enzymes play important roles in two stages of cancer biology: first, in limiting aldehyde genotoxicity in non-malignant cells to prevent malignant transformation, and, second, in shaping the progression and outcome of established cancers, where aldehyde burden and detoxification capacity become frequently dysregulated. To keep our review focused on detoxification enzymes, we direct readers to other comprehensive reviews on the multiple DNA repair pathways that resolve aldehyde-DNA lesions [13,14], and highlight only the specific repair pathways that act in concert with aldehyde detoxification.

## 2. Aldehyde Detoxification Safeguards Genome Integrity Against Cancer Initiation

The human genome encodes 19 ALDH genes [15,16], 15 of which catalyze the nicotinamide adenine dinucleotide (NAD^+^)-dependent oxidation of aldehydes to their corresponding carboxylic acids. In addition to these ALDH enzymes, ADH5, also known as S-nitrosoglutathione reductase (GSNOR), plays a physiological role in formaldehyde detoxification [17,18,23,24]. The glyoxalase system, consisting of GLO1 and HAGH (GLO2), detoxifies the dicarbonyl aldehydes methylglyoxal and glyoxal [19]. The structure, physiological function and expression of these aldehyde detoxification enzymes have been covered in detail by recent reviews [19,25,26,27], and is only discussed briefly here. In this section, we instead focus on the specific detoxification enzymes with evidence linking their detoxification activity to the maintenance of genome integrity in mammalian cells. Where available, we prioritize studies in mouse models, in which conserved murine orthologues [16] of human aldehyde detoxification enzymes provide a tractable genetic system to assess both somatic genome integrity and cancer formation in vivo. We organize the enzymes into three groups based on the strength of evidence linking each enzyme to genome integrity. First, ALDH2, ALDH1B1, ADH5, the ALDH3 family, ALDH9A1, and the glyoxalases (GLO1 and HAGH) have direct genetic evidence in mouse models and/or human genetic syndromes linking enzyme loss to elevated DNA damage and predisposition to cancer; these enzymes are discussed in detail. Second, the ALDH1A family and ALDH4–ALDH8 act on known aldehyde substrates, but direct evidence linking them to genome integrity remains limited; these enzymes are summarized briefly. The remaining four ALDH enzymes do not act on free aldehyde substrates and are not discussed further: ALDH1L1 and ALDH1L2 act on 10-formyltetrahydrofolate in one-carbon metabolism, ALDH18A1 functions in proline biosynthesis, and ALDH16A1 is a catalytically inactive pseudoenzyme.

### 2.1. ALDH2

ALDH2 is a homotetramer localized to the mitochondrial matrix, where it catalyzes the NAD^+^-dependent oxidation of acetaldehyde to acetate [28,29]. ALDH2 has an exceptionally low Km for acetaldehyde (<1 µM), approximately 900-fold lower than the cytosolic isozyme ALDH1, making it the dominant enzyme for acetaldehyde detoxification at physiological concentrations [30]. ALDH2 also oxidizes other reactive aldehydes generated by lipid peroxidation, including 4-hydroxynonenal (4-HNE) [31], malondialdehyde (MDA) [32], and acrolein [32], as well as longer aliphatic (C3–C12 length) and aromatic aldehydes [33,34], positioning it as a broad-spectrum aldehyde detoxification enzyme. A common single-nucleotide polymorphism, ALDH2*2 (rs671, Glu504Lys), is carried by approximately 540 million people of East Asian descent and underpins the acute flushing reaction after alcohol ingestion [35,36,37]. The variant impairs ALDH2 activity, so that acetaldehyde generated from ethanol by alcohol dehydrogenases accumulates rather than being cleared. The polymorphism disrupts the binding of the NAD^+^ cofactor within the active site to impair its enzymatic activity [38,39]. Due to ALDH2’s structure as a tetramer, the incorporation of even a single mutant subunit impairs the enzymatic activity [40] and protein turnover [41] of the entire complex, explaining its dominant inheritance pattern.

#### 2.1.1. ALDH2 Defends Against Genotoxic Acetaldehyde from Alcohol Consumption

IARC has classified alcoholic beverages as a Group 1 human carcinogen [42], supported by both epidemiology studies [43,44] and ethanol exposure studies in rodent models that promote carcinogenesis [45]. Epidemiological studies, predominantly in East Asian populations where the ALDH2*2 allele is common, have established that ALDH2 deficiency is associated with markedly increased alcohol-related cancer risk in the aerodigestive tract, including esophageal squamous cell carcinoma, head and neck squamous cell carcinoma, and gastric cancer [46,47]. These cancer associations are strongly potentiated by alcohol consumption, where, in the absence of functional ALDH2, acetaldehyde accumulates following ethanol ingestion to cause DNA damage [45]. This is demonstrated by direct reactivity between acetaldehyde and DNA to form several adducts, including *N*^2^-ethylidenedeoxyguanosine (or the reduced adduct form *N*^2^-ethyldeoxyguanosine) [48] and 1,*N*^2^-propanodeoxyguanosine, which can further react with deoxyguanosine on the opposite strand to form an interstrand crosslink [48,49] or lysine residues in histones to form DNA–protein crosslinks [50,51]. In vivo, ethanol-fed *Aldh2*^−/−^ mice accumulate greater levels of *N*^2^-ethylidene-deoxyguanosine DNA adducts in the liver [52], stomach [53], and esophagus [54] compared to wild-type controls. Similarly, humans carrying the ALDH2*2 polymorphism with high alcohol consumption had significantly higher acetaldehyde-DNA adducts in blood DNA than those with fully active ALDH2 [55].

Downstream from ALDH2, DNA repair defends against ethanol- and acetaldehyde-induced DNA damage. One such DNA repair mechanism, the Fanconi anemia pathway, is critical for resolving acetaldehyde-induced ICLs [56]. In vertebrate cell lines, the genetic deletion of the FA pathway confers marked hypersensitivity to acetaldehyde-induced growth suppression [20,57,58]. The repair of ICLs by the FA pathway involves the coordinated activity of multiple protein complexes encoded by least 23 FA genes (Fanconi anemia complementation group A–X (*FANCA–FANCX*)) [59]. The FANCM-containing anchor complex recognizes and binds the ICL at a stalled DNA replication fork to promote the assembly of a FA core complex, which monoubiquitinates the FANCI-FANCD2 heterodimer (ID2 complex) to trigger its activation. Monoubiquitinated ID2 complexes form a clamp around the DNA at the site of the ICL and recruit the scaffold protein SLX4 (FANCP), which serves as the central docking platform that coordinates the structure-specific endonucleases that incise the DNA backbone adjacent to the ICL. This creates DNA lesions recognized and repaired downstream by homologous recombination and translesion synthesis. For more mechanistic insights into the FA pathway, we direct readers to other comprehensive reviews [60,61].

ALDH2 and the FA pathway cooperate in mammalian cells to defend against acetaldehyde-induced DNA damage. The requirement for both mechanisms is revealed in mice carrying the combined genetic deletion of *Aldh2* and *Fancd2*, where the ingestion of 15–20% ethanol in water for 10 days increased phosphorylated H2A histone family member X (γH2AX) and severely impaired hematopoiesis in *Aldh2*^−/−^ *Fancd2*^−/−^ mice but not in single knockouts or wild-type mice [20]. Indeed, a single intraperitoneal injection of ethanol to *Aldh2*^−/−^ *Fancd2*^−/−^ mice was sufficient to elevate red cell micronuclei (a marker of chromosome breaks) by 12-fold compared to ethanol-challenged *Aldh2*^−/−^ single-knockout mice, and approximately 20-fold compared to *Fancd2*^−/−^ and wildtype (WT) mice [62]. Metaphase fluorescence in situ hybridization (FISH) on the bone marrow cells from ethanol-challenged mice showed significantly more chromosome aberrations and sister chromatid exchanges, indicative of increased homologous recombination repair against the DNA double strand breaks that result from a loss of the FA-mediated repair of acetaldehyde-DNA lesions [57]. In addition to hematopoietic sensitivity to ethanol, increased teratogenicity was observed in *Aldh2*^−/−^ *Fancd2*^−/−^ mice exposed to ethanol in utero, presenting as exencephaly and eye defects [20], recapitulating features of fetal alcohol syndrome [63].

#### 2.1.2. Detoxification of Endogenous Aldehydes Protects Against HSC Loss and Leukemia

Beyond acetaldehyde from alcohol, ALDH2 also clears endogenous aldehydes generated through normal metabolism. *Aldh2*^−/−^ *Fancd2*^−/−^ mice, in the absence of exogenous alcohol exposure, spontaneously develop elevated DNA damage in hematopoietic tissues [20,57,62,64], with increased γH2AX, chromosome aberrations, sister chromatid exchanges and red cell micronuclei. This endogenous genotoxicity causes a severe depletion of hematopoietic stem cells (HSCs), with a 638-fold reduction in long-term HSCs compared to WT mice [62] and a reconstitution deficit [57]. The surviving HSCs exhibited a significant activation of the p53 transcriptional network, associated with an increased expression of apoptosis-promoting gene *Bax* [64], and cleaved caspase 3 protein level [57], and myeloid lineage differentiation bias, characteristic of stressed [65,66] and aged HSCs [67,68,69]. This potent p53 response is a major driver of the severe attrition and functional decline of HSCs in *Aldh2*^−/−^ *Fancd2*^−/−^ mice, shown by inactivating TP53 in *Aldh2*^−/−^ *Fancd2*^−/−^ *Trp53*^−/−^ mice leading to near-complete rescue in HSC number, reconstitution efficiency, and the suppression of HSC aging features [57,64]. However, this apparent rejuvenation of HSCs comes at the cost of accelerated leukemogenesis. Whereas *Aldh2*^−/−^ *Fancd2*^−/−^ mice develop acute leukemia and thymoma by 16–24 weeks of age [62], *Aldh2*^−/−^ *Fancd2*^−/−^ *Trp53*^−/−^ mice develop leukemia by 6–8 weeks of age (unpublished observation). Overall, integrating the spontaneous phenotype of *Aldh2*^−/−^ *Fancd2*^−/−^ mice reveals that endogenous aldehydes cleared by ALDH2 are a constant genotoxic threat to hematopoiesis and a potent driver of leukemogenesis. Outside the hematopoietic compartment, *Aldh2*^−/−^ mice exhibit mammary ductal epithelial hyperproliferation, elevated oxidative stress and DNA damage signaling, and enhanced mammary stem cell features, in the absence of alcohol exposure [70]. Whether these proliferative changes progress to cancer remains to be determined.

#### 2.1.3. ALDH2 and FA DNA Repair Forms a 2-Tier Protection Against Aldehyde Genotoxicity

The addition of ALDH2 deficiency to FA pathway loss in mice is sufficient to reproduce the major features of the human FA disease: progressive bone marrow failure, HSC attrition, and predisposition to acute leukemia. This is in stark contrast to single-FA-gene knockout mice, which exhibit only mild HSC defects and lack overt bone marrow failure or leukemia development [71,72,73,74]. These mice’s genetic data established a 2-tier model of cellular protection against aldehyde genotoxicity: the first tier comprising enzymatic detoxification by ALDH2 that prevents aldehyde-DNA damage from occurring, and the second tier comprising DNA repair by the FA pathway that resolves damage escaping detoxification [7]. This model, defined in mice, is supported by human genetic evidence. East Asian FA patients carrying the ALDH2*2 polymorphism exhibited significantly accelerated progression to bone marrow failure and leukemia compared to FA patients with wild-type ALDH2 [75,76]. The greater severity of FA in humans than in single-knockout mice may further reflect a higher endogenous aldehyde burden between species; notably, humans have higher circulating formaldehyde levels than mice [77]. A key question arising from the spontaneous phenotype of *Aldh2*^−/−^ *Fancd2*^−/−^ mice is the identity of the endogenous aldehyde(s) responsible for the observed genome instability. Although ALDH2 is best known for detoxifying acetaldehyde, ALDH2-deficient mice do not accumulate acetaldehyde-DNA adducts without alcohol consumption [52,53,54], suggesting other endogenous aldehydes could be drivers of genotoxicity in *Aldh2*^−/−^ *Fancd2*^−/−^ mice. Indeed, ALDH2 has been shown to play an essential role in the clearance of endogenous formaldehyde [23,24,78], discussed together with ADH5 in a later section.

### 2.2. ALDH1B1

ALDH1B1 is the closest paralog of ALDH2, sharing 75% sequence identity and the same mitochondrial matrix localization [79]. Like ALDH2, ALDH1B1 oxidizes acetaldehyde, although with substantially lower affinity (Km 30–55 µM versus <1 µM for ALDH2) [15,80]. ALDH1B1 also exhibits substrate activity towards medium-chain aldehydes (hexanal, nonanal), the short-chain propionaldehyde and aromatic benzaldehyde [80], retinaldehyde [81], and 4-HNE [81,82]. ALDH1B1 is highly expressed in the small and large intestine and the liver [80], and contributes to ethanol catabolism in vivo [83,84]. Several human polymorphisms reduce ALDH1B1 catalytic activity and have been associated with altered alcohol consumption behavior, paralleling the better-known ALDH2*2 variant [81,85].

The evidence supporting a role for ALDH1B1 in protection against acetaldehyde genotoxicity and tumorigenesis comes from mouse studies. Chronic oral administration of 20% ethanol for 12 months to *Aldh1b1*^−/−^ mice resulted in elevated plasma acetaldehyde and intestinal crypt hyperproliferation [83]. Both wild-type and *Aldh1b1*^−/−^ mice developed comparable numbers and sizes of ethanol-induced intestinal tumors and showed similar elevation of γH2AX in intestinal epithelium, but the tumors arising in *Aldh1b1*^−/−^ mice exhibited high-grade dysplasia and locally invasive adenocarcinoma, whereas wild-type tumors remained low-grade adenomas [83]. The liver of *Aldh1b1*^−/−^ mice exhibited sex-dependent changes, with increased hepatocyte proliferation in *Aldh1b1*^−/−^ compared to wild-type male mice, which was further exacerbated by chronic ethanol consumption [86]. Hepatocellular carcinoma developed in one (of eight) male *Aldh1b1*^−/−^ mice following chronic ethanol administration [86].

Subsequent studies from the same group placed ALDH1B1 within a 2-tier protective framework with mismatch repair (MMR), analogous to that established for ALDH2 and the FA pathway in hematopoiesis. MMR corrects base–base mismatches and insertion/deletion loops, principally those generated during DNA replication, through the MutSα (MutS homolog 2, 6 (MSH2, MSH6)) and MutLα (MutL homolog 1 (MLH1), postmeiotic segregation increased 2 (PMS2)) complexes [87]; germline loss of an MMR gene underlies Lynch syndrome and its predisposition to colorectal and other cancers [88]. Using an intestinal epithelial conditional-knockout of *Msh2* (Msh2-LS [89]) to model Lynch syndrome, the long-term administration of 20% ethanol markedly accelerated large intestinal tumorigenesis, with 65% of MMR-deficient mice developing tumors following ethanol treatment versus 4% when treated with water, highlighting the requirement for MMR in the suppression of alcohol-driven intestinal tumorigenesis [90]. A follow-up study generated compound mutants combining intestinal MMR-deficiency (Msh2-LS model) with either constitutive or intestinal-restricted *Aldh1b1* inactivation [91]. Both compound models did not develop spontaneous tumors but exhibited accelerated intestinal tumorigenesis following oral ethanol exposure, with colonic adenomas and adenocarcinomas forming with an average onset of 4.5 months. In contrast, ethanol exposure in mice singly deficient in MMR or ALDH1B1 did not develop tumors by 12 months. Colonic crypts from the double-knockout mice exhibited elevated γH2AX, p53, and cleaved caspase 3, consistent with elevated genotoxic stress.

The functional cooperation between ALDH1B1 and MMR is biochemically unexpected. MMR has not been previously implicated in the repair of aldehyde-DNA lesions: clustered regularly interspaced short palindromic repeats (CRISPR) screens for genes that sensitize cells to aldehydes did not identify MMR components [92,93], and, although a parallel screen identified exonuclease 1 (EXO1), a component in multiple repair pathways, including MMR, as protective against formaldehyde [94], EXO1 deficiency does not sensitize cells to acetaldehyde [20]. In yeast and mammalian cells, MMR loss was likewise dispensable for acetaldehyde sensitivity and mutagenesis [58,95]. The acceleration of ethanol-driven tumorigenesis in *Msh2*/*Aldh1b1* compound mutants therefore likely reflects a downstream role for MMR in resolving the secondary genotoxic consequences of acetaldehyde-DNA lesions rather than the direct repair of the lesions themselves. Together, these studies establish ALDH1B1 as a tier 1 enzymatic defense against alcohol-derived acetaldehyde in the intestine, with MMR providing a tier 2 DNA repair that limits the malignant consequences of acetaldehyde-induced DNA damage. The same 2-tier conceptual framework therefore extends from hematopoietic tissues (ALDH2 with the FA pathway) to the intestine (ALDH1B1 with MMR).

### 2.3. ADH5

ADH5 is a ubiquitously expressed cytosolic enzyme with two distinct physiological functions. First, ADH5 is the principal enzyme responsible for clearing formaldehyde, a ubiquitous and highly reactive aldehyde classified under IARC as a Group 1 human carcinogen [8,96]. Formaldehyde initially conjugates with reduced glutathione (GSH) to form S-hydroxymethylglutathione, which ADH5 oxidizes in an NAD^+^-dependent reaction to S-formylglutathione [17], with subsequent hydrolysis by S-formylglutathione hydrolase (ESD) to yield non-toxic formate with the regeneration of GSH [18]. Formate can then re-enter one-carbon metabolism to fuel nucleotide biosynthesis [9,97]. Second, ADH5 functions as S-nitrosoglutathione (GSNO) reductase, degrading the spontaneous nitric oxide–glutathione conjugate GSNO to hydroxylamine and glutathione disulphide [98]. This second activity positions ADH5 as a regulator of cellular nitric oxide level and protein nitrosylation, a post-translational modification involving the covalent addition of nitric oxide to cysteine thiols [99].

#### 2.3.1. Detoxification of Genotoxic Formaldehyde

Formaldehyde reacts with DNA to form monoadducts, interstrand crosslinks and DNA–protein crosslinks, which can disrupt DNA replication and transcription, and cause mutagenesis [13,14]. Beyond direct genotoxicity, elevated formaldehyde can also indirectly promote oxidative DNA damage by competing for GSH that would otherwise sequester reactive oxygen species [100]. Interestingly, elevated formaldehyde has also been shown to induce the ubiquitin-mediated proteasomal depletion of BRCA2, which in cells harboring pre-existing BRCA heterozygosity can result in functional haploinsufficiency [101]. ADH5 has a clear role in defending against formaldehyde from environmental exposure, either directly [102,103] or via methanol catabolism [23,104,105]. However, mammalian ADH5 plays an equal if not greater role in detoxifying the substantial formaldehyde burden generated by cellular metabolism itself. Using liquid chromatography–mass spectrometry (LC–MS) to quantify formaldehyde-DNA adducts as stable biomarkers, the endogenous formaldehyde burden was initially characterized by inhalation-exposure studies in rodents [106,107]. The inhalation of isotope-labeled formaldehyde resulted in isotopic formaldehyde-DNA adducts only in the nasal epithelium, whereas endogenous formaldehyde-DNA adducts were detected in both nasal epithelium and distant tissues that were not directly exposed. The magnitude of endogenous formaldehyde production was subsequently revealed in mouse models lacking both ADH5 and ALDH2 [23]. Without any exogenous formaldehyde exposure, *Aldh2*^−/−^ *Adh5*^−/−^ mice harbored a 100-fold increase in formaldehyde-DNA adducts [23], revealing both the magnitude of metabolic formaldehyde production and that ALDH2 was needed to detoxify formaldehyde in vivo. Many biochemical reactions that take place in mammalian cells can generate formaldehyde, revealed mainly through in vitro and cell line studies, including the enzymatic demethylation of proteins, nucleic acids and metabolites [108,109], folate metabolism [9,110], and mitochondrial metabolism [10,111]. The physiological and pathological factors that determine endogenous formaldehyde levels in vivo remain an active area of research.

The accumulation of endogenous formaldehyde in *Aldh2*^−/−^ *Adh5*^−/−^ mice results in significant genome instability, as evidenced by increased red cell micronuclei, sister chromatid exchanges, and the upregulation of p53 network and DNA repair genes in the single-cell RNA sequencing of hematopoietic stem and progenitor cells (HSPCs) [23]. The whole-genome sequencing of ex vivo expanded HSPC clones from these mice revealed elevated mutational burden enriched for T to A and T to G transversions, resembling the COSMIC SBS40 signature present in many human cancers [23]. Even in *Adh5*^−/−^ mice, where endogenous formaldehyde is elevated but to a lesser degree than *Aldh2*^−/−^ *Adh5*^−/−^ mice, the FA DNA repair pathway was still required for genome maintenance. This is evidenced by combined deletions of ADH5 and the FA pathway in cell lines and mice, with both genetic models exhibiting elevated chromosome breaks and elevated γH2AX [21,77,112]. Beyond the FA pathway, ADH5 deficiency also synergizes with loss of Cockayne syndrome B protein (CSB) [113], a component of transcription-coupled nucleotide excision repair, and with DNA polymerase theta (Polθ)-mediated end joining, which repairs formaldehyde-induced DPCs [114,115]. These genetic interactions establish that endogenous formaldehyde engages multiple DNA repair pathways and that ADH5 functions as the critical first line of defense, whose failure exposes the genome to a diversity of formaldehyde-induced lesions.

#### 2.3.2. Tissue Dysfunction and Cancer Following Failed Formaldehyde Detoxification

The genotoxic consequences of formaldehyde on tissue homeostasis are severe. Both *Aldh2*^−/−^ *Adh5*^−/−^ [23,24,116] and *Adh5*^−/−^ *Fancd2*^−/−^ mice [105] exhibit failure to thrive and die within 1–2 months following birth. *Adh5*^−/−^ *Fancd2*^−/−^ mice die from bone marrow failure driven by a 952-fold reduction in long-term HSC frequency leading to a collapse in hematopoiesis. In contrast, *Aldh2*^−/−^ *Adh5*^−/−^ mice exhibited only a 3- to 4-fold loss in HSC number, which could maintain blood production, which indicates that the cause of their early lethality lies outside hematopoiesis and remains incompletely characterized. The cancer consequences of formaldehyde are evident in both mouse models. *Aldh2*^−/−^ *Adh5*^−/−^ mice surviving beyond 7 months develop acute leukemia and liver cancer, while *Adh5*^−/−^ *Fancd2*^−/−^ mice transplanted with wild-type bone marrow to rescue acute hematopoietic failure develop acute leukemia and hepatocellular carcinoma. The genome-protective role of ADH5 and ALDH2 is recapitulated in humans, where children born with a digenic loss of ADH5 and the ALDH2*2 polymorphism present with AMeD syndrome (aplastic anemia, mental retardation and dwarfism), phenocopying the mouse model with bone marrow failure and acute myeloid leukemia (AML) [23,24,78]. The resemblance to Fanconi anemia is consistent with a common genotoxic aldehyde driver of disease in both inherited syndromes, and demonstrates the essential roles of ALDH2 and ADH5 in protecting the hematopoietic cells from endogenous formaldehyde.

#### 2.3.3. Preventing Clonal Hematopoiesis Arising from HSC Attrition

Beyond protecting against acute leukemia, ADH5 also plays a critical role in preventing clonal hematopoiesis, a common age-associated pre-leukemic condition in which blood production is dominated by clonally expanded HSCs and associated with an increased risk of hematological malignancies [117,118] and cardiovascular disease [119]. To study the specific requirement for formaldehyde detoxification in the hematopoietic compartment, a recent study generated hematopoietic-specific *Adh5* conditional knockout mice (*Adh5*^c/−^ *Fancd2*^−/−^ *Vav1-iCre*) [77], restricting ADH5 loss to the blood compartment while preserving systemic formaldehyde clearance. The HSC depletion in these conditional mice was less severe than in constitutive knockouts, conferring longer survival through sustained blood production. Whole-genome sequencing combined with computational clonal inference (SCIFER [120]) was used to mathematically model the number of HSC clones giving rise to peripheral blood cells. While aged control mice (27–44 weeks) maintained polyclonal hematopoiesis, *Adh5*^c/−^ *Fancd2*^−/−^ *Vav1-iCre* mice rapidly transitioned from polyclonal at 5 weeks of age to monoclonal hematopoiesis by 25–37 weeks of age, with blood production traceable to a single blood-forming HSC [77]. Strikingly, the dominant clones lacked any identifiable driver mutations, demonstrating that chronic endogenous formaldehyde can drive stochastic stem cell attrition to give rise to clonal hematopoiesis without obvious positive selection. This highlights an alternative path to the emergence of clonal hematopoiesis distinct from the canonical acquisition of mutations conferring positive selective advantage [121,122,123]. The same pattern of accelerated, driverless clonal hematopoiesis was observed in Fanconi anemia patients [77]. These findings position ADH5 not only as a guardian against formaldehyde-induced cancer but also as a critical determinant of HSC pool maintenance, where its loss accelerates the trajectory toward clonal hematopoiesis and eventual bone marrow failure.

#### 2.3.4. Protection Against S-Nitrosylation Mediated Genotoxicity

Outside of formaldehyde detoxification, ADH5’s dual GSNO reductase role has been implicated in genome instability in tissue-specific contexts. In the liver, ADH5 deficiency causes GSNO accumulation, elevated protein S-nitrosylation, and spontaneous hepatocellular carcinoma [124,125]. This hepatocarcinogenesis is prevented by the co-deletion of inducible nitric oxide synthase (iNOS), indicating that nitric oxide, rather than formaldehyde, drives liver tumor formation in this model. The proposed mechanism involves the S-nitrosylation-mediated inactivation of DNA repair proteins: O6-alkylguanine-DNA alkyltransferase (AGT/MGMT), 8-oxoguanine DNA glycosylase (OGG1), apurinic/apyrimidinic endodeoxyribonuclease 1 (APE1), and DNA-dependent protein kinase catalytic subunit (DNA-PKcs) have all been identified as targets of S-nitrosylation [126], and MGMT inactivation in *Adh5*^−/−^ liver leads to the accumulation of mutagenic O6-methylguanine lesions. The tissue specificity of this mechanism is underscored by the observation that iNOS co-deletion does not rescue the hematopoietic failure of *Adh5*^−/−^ *Fancd2*^−/−^ mice. ADH5 thus illustrates how a single enzyme can protect the genome through fundamentally different mechanisms in different tissues: formaldehyde clearance in hematopoietic cells, and nitric oxide regulation in hepatocytes.

### 2.4. ALDH3 Family

The ALDH3 family comprises four members (ALDH3A1, ALDH3A2, ALDH3B1, and ALDH3B2) with complementary but overlapping functions in the oxidation of medium- and long-chain fatty aldehydes (C6–C20), products of lipid peroxidation, sphingolipid degradation (hexadecanal, trans-2-hexadecenal), and omega-6 fatty acid metabolism [15]. ALDH3A2 (fatty aldehyde dehydrogenase, FALDH) is the best characterized family member: its loss causes Sjögren–Larsson syndrome (SLS), an inborn error of lipid metabolism characterized by ichthyosis, spastic diplegia, and cognitive impairment [127]. The underlying mechanism of disease results from toxic fatty aldehyde accumulation, causing membrane disruption and oxidative stress in skin and brain [128]. SLS is not associated with cancer predisposition, and DNA damage has not been assessed in this context. ALDH3 deficiency has also been associated with sensitization to ferroptosis in AML and ovarian cancer cells, where unprocessed lipid aldehydes overwhelm the glutathione antioxidant axis [129,130,131,132]. The physiological relevance of ferroptosis-associated genotoxicity is uncertain given the dominant membrane damage in this cell death modality.

#### Detoxification of Genotoxic Fatty Aldehydes

There is now substantial evidence that the fatty aldehydes cleared by ALDH3 enzymes are bona fide genotoxins that directly damage DNA. The α-, β-unsaturated aldehydes generated during lipid peroxidation, particularly 4-HNE, acrolein, crotonaldehyde, and MDA, react with DNA bases to form a diverse spectrum of mutagenic adducts [133,134]. 4-HNE, the most abundant product of omega-6 polyunsaturated fatty acid peroxidation, reacts with deoxyguanosine to form 1,*N*^2^-propano-2′-deoxyguanosine (HNE-dG) adducts, which have been detected in human and rodent tissue DNA [135]. Alternatively, the oxidation of 4-HNE to 2,3-epoxy-4-hydroxynonanal yields etheno adducts, including 1,*N*^6^-ethenodeoxyadenosine (εdA) [136,137]. The DNA adducts arising from these unsaturated aldehydes are mutagenic, with acrolein- and crotonaldehyde-DNA adducts inducing base substitutions (predominantly G to T transversions) and frameshift mutations [133]. HNE has been shown to preferentially induce G:C to T:A transversions at codon 249 of human *TP53* gene, a known mutational hotspot in human cancers [138]. Critically, acrolein- and HNE-derived adducts can also form interstrand DNA crosslinks [139,140]. MDA, one of the most abundant endogenous lipid peroxidation products, reacts with guanine to form the pyrimidopurinone adduct M_1_dG, which is detected in healthy human tissues at levels of 1–120 per 10^8^ nucleotides [134,141], and induces both frameshifts and base substitutions [142]. These adducts are collectively repaired by NER [143,144], translesion synthesis involving Y-family polymerases (pol κ, pol ι) [145], and mismatch repair [146], establishing lipid aldehydes as a class of endogenous genotoxin requiring multiple DNA repair pathways.

Genetic evidence reveals a role for ALDH3 family enzymes for genome protection in somatic non-malignant cells. To define relevant genotoxic aldehydes that drive squamous cell carcinoma, a common malignancy in FA, a genetic study systematically inactivated all four ALDH3 enzymes in immortalized keratinocytes from humans and mice to reveal the activation of the FA pathway through FANCD2 monoubiquitylation [147]. Furthermore, the combined inactivation of all four ALDH3 enzymes with the FA pathway (*FANCA* deletion) resulted in spontaneous growth deficit, elevated γH2AX and S/G2 phase cell cycle arrest. FA-deficient keratinocytes were also more sensitive to exogenous 4-HNE treatment than FA-competent cells [147]. This study highlights the ALDH3 family as tier 1 defense enzymes against endogenous genotoxic fatty aldehydes in keratinocytes, with the FA pathway providing tier 2 repair. This finding has immediate clinical significance for understanding FA-associated predisposition for squamous cell carcinomas, revealing a distinct endogenous aldehyde threat from lipid peroxidation, requiring ALDH3 family enzymes for genome protection to prevent malignant transformation.

### 2.5. ALDH9A1

ALDH9A1 is best characterized for its role in carnitine biosynthesis (oxidizing γ-trimethylaminobutyraldehyde to γ-butyrobetaine) [148,149] and γ-aminobutyric acid (GABA) metabolism (oxidizing 4-aminobutyraldehyde to GABA) [150], but has broad substrate specificity spanning aminoaldehydes, betaine aldehyde, short-chain aliphatic aldehydes (acetaldehyde and hexanal), and aromatic aldehydes (benzaldehyde and 3,4-dihydroxyphenylacetaldehyde) [149,151].

#### Genome Instability and Cancer from Loss of ALDH9A1 and the FA Pathway

As well as its physiological roles in cell metabolism, genetic evidence in cell lines and mice highlights a role for ALDH9A1 in maintaining genome stability within the FA pathway [112]. ALDH9A1 emerged as the top synthetic lethal gene when inactivated in FANCD2-deficient human Jurkat T cell line in a metabolism-focused CRISPR screen. A subsequent analysis of *ALDH9A1*^−/−^ *FANCD2*^−/−^ Jurkat cells revealed spontaneous growth deficit with elevated chromosome breaks, increased p53-binding protein 1 (53BP1) and γH2AX foci, and apoptosis, all indicative of increased endogenous DNA damage. In primary human umbilical cord CD34^+^ cells, the combined genetic inactivation of ALDH9A1 and FANCD2 reduced clonal expansion and survival [112]. In contrast to cell line models, *Aldh9a1*^−/−^ *Fanca*^−/−^ mice exhibited intact hematopoiesis with mild reduction in HSPC populations. Interestingly, aged *Aldh9a1*^−/−^ *Fanca*^−/−^ mice (9–24 months) developed an increased frequency of ovarian tumors, which were not observed in single-knockout controls. In contrast, *Aldh2*^−/−^ *Fancd2*^−/−^ and *Adh5*^−/−^ *Fancd2*^−/−^ mice develop a severe attrition of HSPCs and acute leukemia. As all three mice models are 2-tier deficient throughout all tissues, the distinct tissue-specific phenotypes could reflect differences in the predominant endogenous aldehydes generated in each tissue, the expression of compensatory ALDH family members that provide metabolic redundancy, or both. Indeed, the synthetic lethality of combined ALDH9A1 and FANCD2 deficiency in Jurkat cells was rescued by the deletion of the polyamine transporter adenosine triphosphatase 13A3 (ATP13A3) [152], implicating polyamine-derived aldehydes as the relevant ALDH9A1 substrates. Polyamine catabolism generates 4-aminobutyraldehyde (the canonical ALDH9A1 substrate) and 3-aminopropanal, which spontaneously decomposes to the potent genotoxin acrolein if not cleared [153]. The ovarian tropism in *Aldh9a1*^−/−^ *Fanca*^−/−^ mice may reflect the elevated polyamine synthesis activity reported in ovarian tissue [154], which would generate substantial aminoaldehydes, requiring ALDH9A1 for clearance.

### 2.6. GLO1 and HAGH

Glyoxalase 1 (GLO1) and hydroxyacylglutathione hydrolase (HAGH, also known as GLO2) detoxify the dicarbonyl aldehydes glyoxal and methylglyoxal (MG) [19]. Glyoxal arises from lipid peroxidation and the degradation of glycated proteins and monosaccharides, while MG arises mainly from the spontaneous degradation of the glycolytic intermediates dihydroxyacetone phosphate and glyceraldehyde-3-phosphate [11]. Similar to ADH5, GLO1 does not act on free dicarbonyls but on their spontaneously formed glutathione conjugates, generating S-glycolylglutathione and S-D-lactoylglutathione respectively, which are subsequently hydrolyzed by HAGH to glycolate and D-lactate with the regeneration of free GSH. Under basal conditions, MG is maintained at low micromolar concentrations by the glyoxalase system. The abnormal accumulation of MG, termed dicarbonyl stress, is a feature of type 2 diabetes, where elevated glycolytic flux overwhelms MG clearance and drives the formation of advanced glycation end products on proteins and DNA [155,156].

#### 2.6.1. Methylglyoxal as an Endogenous Genotoxin

MG forms a range of DNA adducts, predominantly with deoxyguanosine, including the *N*^2^-modified adduct *N*^2^-(1-carboxyethyl)-2′-dG and the cyclic imidazopurinone adduct 1,*N*^2^-(1,2-dihydroxy-2-methyl)ethano-dG [157,158]. MG-dG adducts have been shown to be elevated in tissues, blood, and urine from humans with type 2 diabetes [158,159]. Site-specific oligonucleotide [160,161] and a shuttle vector study [162] in human fibroblasts have established that MG-dG adducts are mutagenic, biased toward G to T and G to C transversions, and are repaired in part by nucleotide excision repair. The genome-wide sequencing of MG-treated *Glo1*-deficient yeast revealed a strand-asymmetric MG-associated mutational signature dependent on the translesion polymerase Rev1, and this signature is enriched across multiple human tumor genome datasets [163], providing evidence that endogenous MG contributes to mutational burden in human cancer.

#### 2.6.2. GLO1 Cooperates with BRCA2 to Suppress Methylglyoxal-Induced Genotoxicity

Genetic evidence that the loss of MG detoxification drives genome instability comes from a study combining GLO1 inactivation with biallelic BRCA2 loss in non-malignant breast epithelial cells [22]. Combined GLO1/BRCA2 inactivation produced spontaneous γH2AX foci and mutagenesis, with further amplification by exogenous MG. Notably, MG also directly compromises tier 2 repair: treatment of cells bearing monoallelic *BRCA2* mutations treated with intracellular-physiological concentrations of MG (300–700 µM) induced the degradation of BRCA2, producing functional BRCA2 haploinsufficiency, replication fork instability, and γH2AX accumulation. Chronic MG exposure in this monoallelic *BRCA2* context elicited a mutational signature characteristic of human cancers harboring biallelic *BRCA2* inactivation. These findings establish a 2-tier model in which GLO1 detoxifies MG to prevent DNA damage (tier 1), and BRCA2-dependent homologous recombination resolves any damage that escapes detoxification (tier 2), with the additional and unusual feature that tier 1 failure can directly compromise tier 2 by depleting BRCA2 itself. The evidence that glyoxalase loss alone drives cancer is more limited. *Glo1* single-knockout zebrafish [164,165] and mice [166] exhibit metabolic dysregulation but no cancer phenotype, possibly reflecting a compensatory upregulation of ALDH activity; however, these studies were not intended for cancer assessment, and animal models combining glyoxalase loss with DNA repair deficiency, analogous to the *Aldh2*^−/−^ *Fancd2*^−/−^ or *Adh5*^−/−^ *Fancd2*^−/−^ mice, have yet to be reported. Whether GLO1 deficiency in vivo phenocopies the synthetic interactions seen in cell-based BRCA2 systems remains an important open question.

### 2.7. Other ALDH Family Members with Limited Evidence for Genome Integrity Roles

The remaining ALDHs have limited direct evidence linking them to genome integrity. The ALDH1A subfamily (ALDH1A1, A2, A3) converts retinaldehyde to retinoic acid, a critical signaling modulator of gene expression and differentiation [167], and has been extensively studied as a marker of cancer stem cells and mediators of therapeutic resistance [168]. The evidence linking ALDH1A enzymes to genome integrity is suggestive but limited to cell line systems. ALDH1A1 knockdown in the platinum-resistant ovarian cancer cell line A2780/CP70 caused spontaneous γH2AX elevation, with reduced protein levels of DNA damage response (phosphorylated checkpoint kinase 1 (CHK1), p21) and DNA repair factors (FANCD2, FANCJ, and X-ray repair cross-complementing 1 (XRCC1)) [169]. The genetic deletion of ALDH1A3 in ovarian cancer cells resulted in elevated sensitivity to retinaldehyde and 4-HNE, and synergized with ataxia telangiectasia mutated (ATM) and ataxia telangiectasia and Rad3 related (ATR) inhibitors in growth suppression [170]. However, *Aldh1a1* knockout mice are developmentally normal without gross stem cell or cancer phenotypes [171], *Aldh1a2* knockout is embryonically lethal without retinoic acid rescue [172,173], and *Aldh1a3* knockout is perinatally lethal due to retinoic acid-signaling defects [174]; genome instability has not been examined in any of these models.

ALDH4A1 (P5CDH) catalyzes the oxidation of L-glutamate-5-semialdehyde derived from proline catabolism [15]. It has been shown to be a transcriptional target of p53, induced in response to genotoxic stress to protect cells from oxidative stress following hydrogen peroxide or UV exposure [175]. ALDH4A1-deficient mice exhibit increased sensitivity to a non-canonical aldehyde substrate 4-HNE [176], but DNA damage markers were not evaluated. ALDH4A1 also functions non-enzymatically as a component of the mitochondrial pyruvate carrier complex, and its loss elevates glycolysis [177], raising the untested possibility of indirect genotoxicity through elevated methylglyoxal flux. ALDH5A1 (SSADH) converts succinic semialdehyde to succinate to feed into the tricarboxylic acid (TCA) cycle [15]. *Aldh5a1*^−/−^ mice exhibited reduced glutathione in the cortex and hippocampus, but ROS, DNA damage markers, and cancer phenotypes have not been assessed [178]. ALDH6A1 (MMSDH) functions in valine and pyrimidine catabolism [15]; one study reported altered ROS levels with overexpression in liver cancer cells but no direct genotoxicity measurements [179]. ALDH7A1 (antiquitin) acts on α-aminoadipic semialdehyde in lysine degradation pathway and on lipid peroxidation products [15]. Its loss causes pyridoxine-dependent epilepsy through aldehyde-mediated vitamin B6 depletion, and it has been implicated in suppressing ferroptosis through the detoxification of 4-HNE and MDA [180], but no studies have examined its role in DNA damage.

ALDH8A1 oxidizes 9-cis-retinaldehyde to 9-cis-retinoic acid [15] and is expressed predominantly in liver and kidney, with low expression in the hematopoietic compartment. Although ALDH8A1 has no established physiological role in genome maintenance, it exhibits formaldehyde-detoxifying activity when ectopically expressed [181] in K562 and HL60 leukemia cells [181]. These leukemic cell lines retain the ability to differentiate, and require the FA pathway to protect against differentiation-induced apoptosis, which is likely driven by formaldehyde generated during histone demethylation accompanying transcriptional reprogramming [182]. While several aldehyde detoxification enzymes (ADH5, ALDH8A1, ALDH4A1, ALDH3B1, and ALDH3B2) reduced this apoptosis when overexpressed in these differentiating cells, ALDH8A1 produced the strongest effect [181]. ALDH8A1 overexpression lowered nuclear formaldehyde, γH2AX and 53BP1 foci, and DPCs during differentiation and restored productive lineage progression, dependent on its catalytic activity. Furthermore, recombinant ALDH8A1 exhibited the direct enzymatic oxidation of glutathione-formaldehyde conjugate S-hydroxymethylglutathione with a substantially lower Km than ADH5. Because ALDH8A1 is not normally expressed in hematopoietic cells, whether it contributes to endogenous formaldehyde detoxification in vivo remains unknown, but these findings nominate enhanced ALDH-mediated formaldehyde clearance as a potential therapeutic strategy in FA.

## 3. Aldehyde Detoxification Shapes Genome Integrity in Established Cancers

In the preceding sections, the consensus from studies of aldehyde detoxification enzymes across diverse non-malignant tissues converges on a need for mammalian cells to protect the genome from both endogenous and exogenous aldehyde-induced genotoxicity. Failure to do so can compromise cellular viability and directly accelerate cancer initiation. Following malignant transformation, does the cancer cell also rely on aldehyde detoxification to maintain genome integrity? Addressing this question requires a more nuanced integration of cellular complexities unique to cancer biology. First, cancers actively exploit genomic instability as a fundamental enabling hallmark to acquire driver mutations during tumor evolution [1]. This is frequently achieved through the inactivation of DNA repair pathways and corruption of DNA damage sensing and response mechanisms, conferring tolerance for elevated genotoxic burden. Second, the rewiring of metabolism and energetics within cancer cells can alter their endogenous production of aldehydes, creating dependencies on aldehyde detoxification enzymes that are not required in the cell of origin. Third, aldehyde detoxification capacity in cancer is frequently dysregulated, through the altered expression of detoxification enzymes as well as increased demand for glutathione, the major aldehyde-sequestering molecule.

Together, these complexities limit broad generalizations on the genome-protective role of aldehyde detoxification enzymes in cancer, and instead require studies on specific cancer types while recognizing that conclusions may not extend across cancer types. To this end, this section reviews the available data on the expression of aldehyde detoxification enzymes in individual cancers, followed by an appraisal of studies from specific cancers on how altered aldehyde production and/or detoxification impacts genome integrity and outcomes. In addition to genome stability, aldehyde detoxification enzymes have been shown to serve as cancer stem cell biomarkers [183,184] and contribute to cell signaling, differentiation, chemoresistance, metastatic potential, and immune evasion. These roles have been comprehensively reviewed elsewhere and are not discussed further here [168,184,185].

### 3.1. Altered Expression of Aldehyde Detoxification Enzymes Across Human Cancers

**Figure 1:**
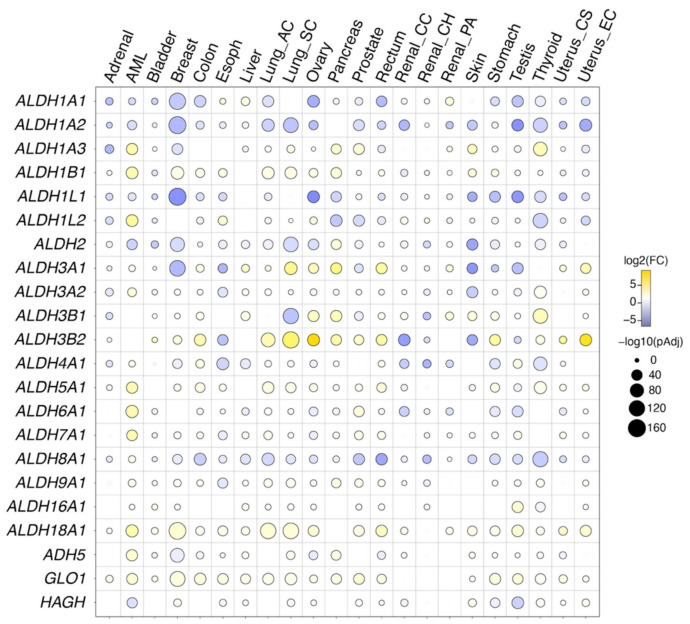
Differential expression of aldehyde detoxification genes across human cancers. Dot matrix showing the expression of 22 aldehyde detoxification genes across 22 cancer types relative to normal tissue. Dot color represents the log2 fold change (log2(FC)) of expression in tumor versus normal tissue, with yellow indicating higher expression in tumor and blue indicating lower expression. Dot size corresponds to statistical significance on a −log10(adjusted *p*-value) scale, where larger dots indicate smaller *p*-values. Data were obtained and plotted using TNMplot v2 [195], which compares RNA-seq expression between tumor and normal tissues using publicly accessible data from the Genomic Data Commons (TCGA/TARGET) and the Genotype-Tissue Expression (GTEx) project. The integrated analysis and visualization presented here are original to this review. Cancer type abbreviations: Esoph, esophageal carcinoma (all subtypes); Lung_AC, lung adenocarcinoma; Lung_SC, lung squamous cell carcinoma; Renal_CC, clear cell renal cell carcinoma; Renal_CH, chromophobe renal cell carcinoma; Renal_PA, papillary renal cell carcinoma; Uterus_CS, uterine carcinosarcoma; Uterus_EC, uterine corpus endometrial carcinoma.

For this review, we performed an original analysis of the differential expression of all 19 ALDH isoforms, ADH5, GLO1, and HAGH across 22 cancer types using the latest available transcriptome data from the TNMplot platform (which aggregates publicly available samples from the GEO, GTEx, TCGA, and TARGET databases) (Figure 1). Our results complement prior studies to reveal that ALDH [25,168,186], ADH5 [187,188,189,190,191] and glyoxalase [192,193,194] enzymes exhibit both up- and downregulation in expression in human cancer. The majority of ALDH family members are downregulated in tumors relative to matched normal tissue. ALDH1A1, ALDH1A2, ALDH2, ALDH4A1 and ALDH8A1 show consistent downregulation across a broad range of tumors, with particularly pronounced reductions in kidney, liver, lung, and colon cancers. In contrast, ALDH1B1 and GLO1 are upregulated in most tumors. A smaller subset of isoforms exhibits divergent patterns across different cancers: ALDH3A1 and ALDH3B2 show dramatic overexpression in cancers of lung, ovary, pancreas and uterus but downregulation in esophagus, renal and skin. ALDH1A3 is upregulated in AML, pancreas, prostate, skin and thyroid cancers, but downregulated in adrenal, breast and uterus cancers. Each isoform thus has a distinct expression signature, and the direction and magnitude of dysregulation varies markedly across cancer types.

**Figure 2:**
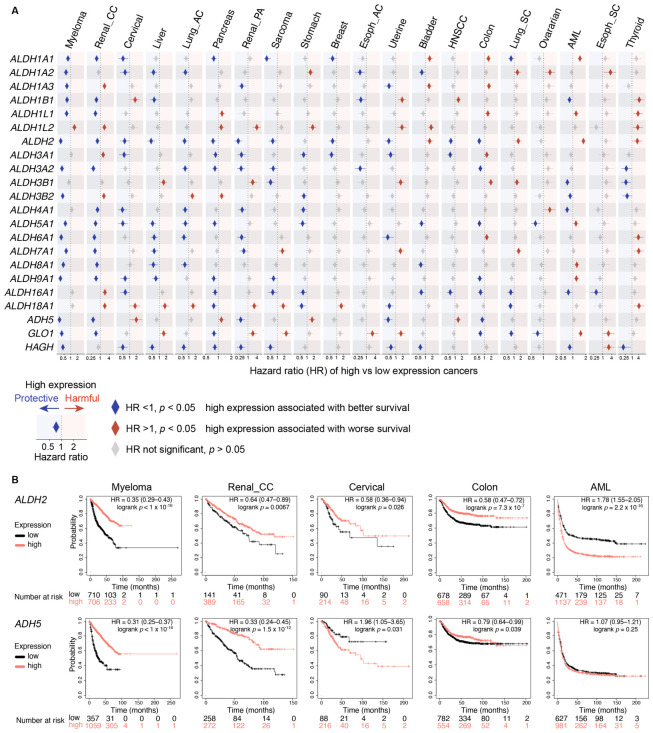
Pan-cancer expression–survival landscape of aldehyde detoxification genes. (**A**) Forest plots showing hazard ratios (HR) and 95% confidence intervals for overall survival by expression level of 22 aldehyde detoxification genes across 20 cancer types. Each diamond represents the HR for high versus low expression, with the cut-off selected to maximize survival difference and statistical significance. Blue diamonds indicate high expression associated with significantly better survival (HR < 1, *p* < 0.05); red diamonds indicate high expression associated with significantly worse survival (HR > 1, *p* < 0.05); grey diamonds indicate non-significant associations (*p* ≥ 0.05). Cancer types are ordered by the predominance of protective associations across the gene panel. Note that x-axis scales differ between cancer types to accommodate variation in HR magnitude. The integrated analysis and visualization presented here are original to this review. Cancer type abbreviations: Renal_CC, clear cell renal cell carcinoma; Cervical, cervical squamous cell carcinoma; Liver, hepatocellular carcinoma; Lung_AC, lung adenocarcinoma; Renal_PA, papillary renal cell carcinoma; Stomach, gastric adenocarcinoma; Esoph_AC, esophageal adenocarcinoma; Uterine, uterine corpus endometrial carcinoma; HNSCC, head and neck squamous cell carcinoma; Lung_SC, lung squamous cell carcinoma; Esoph_SC, esophageal squamous cell carcinoma; Thyroid, thyroid carcinoma. The forest plot was generated with the assistance of Claude (Anthropic) Opus 4.7 using Python 3.12.3 with openpyxl 3.1.5, numpy 2.4.4, and matplotlib 3.10.8. (**B**) Representative Kaplan–Meier survival curves for ALDH2 and ADH5 across five cancer types illustrating different patterns between expression and survival. In myeloma, clear cell renal cell carcinoma, and colon adenocarcinoma, high ALDH2 and ADH5 expression are protective. In cervical squamous cell carcinoma, high ALDH2 expression is protective but high ADH5 expression is harmful. In AML, high ALDH2 is harmful in contrast to other cancers. Survival data for forest plot and Kaplan–Meier curves were obtained from KMPlotter [196] that correlate survival with RNA-seq expression data for all cancer types except myeloma, pancreas, AML, and colon, where microarray expression data were used to maximize cohort size. HR, 95% CI, and log-rank *p*-values are shown for each comparison.

The biological significance of these expression changes is reflected in cancer prognosis [168]. We performed an original systematic Kaplan–Meier survival analysis of all 22 enzymes across 20 cancer types using KMPlotter [196], integrating expression and survival data from GEO, EGA, and TCGA (Figure 2). For the majority of aldehyde detoxification enzymes, high expression is significantly associated with better overall survival across multiple cancer types. Myeloma emerges as the cancer most broadly dependent on aldehyde detoxification capacity, with 16 of 22 enzymes showing significant protective associations. Renal clear cell carcinoma, cervical, and liver cancer also show strong net protective signals. In contrast, AML and thyroid cancers exhibit net harmful signal for the ALDH/ADH family overall; for example, high ALDH2 expression is significantly associated with worse overall survival in both cancers, a reversal compared to many other cancer types examined. This reversal suggests that the role of aldehyde detoxification is not fixed but context-dependent, shaped by the metabolic and genetic landscape of the tumor. Expression-survival correlations alone, however, cannot establish whether dysregulated detoxification actively drives genome instability or merely reflects it. In the following sections, we examine the specific cancers where this question has been addressed mechanistically.

### 3.2. Elevated Endogenous Aldehydes in Esophageal Cancer and AML

While the transcriptome analysis of human cancers reveals widespread dysregulation in the expression of aldehyde detoxification enzymes, only a few studies have been conducted to characterize the levels of endogenous aldehydes in cancers. In esophageal cancers, evidence suggests ALDH3 enzymes play a role in limiting fatty aldehyde accumulation and genotoxicity. A comprehensive analysis [136] of esophageal adenocarcinoma (EAC) with cancer-adjacent squamous revealed a reduced expression of *ALDH3A1*, *ALDH3A2* as a hallmark of EAC compared to normal squamous esophageal epithelium, consistent with data from TNMPlotter (Figure 1). Using targeted LC–MS, 42 aldehydes, including short-chain aldehydes (formaldehyde, acetaldehyde, 4-HNE, 1-butenal) and medium-chain alkanals (nonanal, decanal, undecanal and dodecanal), were significantly enriched in EAC tissue [136]. Furthermore, aldehyde-DNA adducts (acetaldehyde-derived adduct and an HNE-derived 1,*N*^6^-εdA adduct) were found both to be significantly elevated in EAC and adjacent squamous tissue compared to normal esophageal mucosa and circulating leukocytes. In particular, increased tissue decanal was linked to poorer overall survival, reduced *ALDH3A2* expression and *TP53* deletion on chromosome 17p. However, *ALDH3A2* and *TP53* genes are both located on chromosome 17p, suggesting that ALDH3A2 loss and the resulting decanal accumulation are collateral consequences of the TP53-targeting 17p arm deletion that characterizes EAC progression, rather than independent drivers of worse outcomes. Nevertheless, this study provides direct evidence that endogenous aldehyde accumulates in human cancer tissue, with confirmed aldehyde-DNA adducts demonstrating genotoxic consequences.

AML provides complementary evidence of elevated endogenous aldehydes in cancer, with mechanistic evidence linking the elevation to oncogenic transformation. Using an enzymatic fluorometric assay, a panel of human AML cell lines harbored elevated endogenous formaldehyde compared to multiple solid cancer cell lines [114]. Increased formaldehyde production was directly induced by oncogenic transformation: constitutive expression of BCR-ABL, FLT3(ITD), or JAK2(V617F) in immortalized non-leukemic murine bone marrow cells (32D) resulted in approximately 40% elevation in endogenous formaldehyde, accompanied by a 2- to 4-fold increase in DPCs. A culture of transformed 32D cells in media depleted of glucose, serine, and glycine reduced formaldehyde levels, DPC accumulation, and γH2AX-positive cell fractions. Treatment with the serine hydroxymethyltransferase 1/2 (SHMT1/2) inhibitor SHIN1 phenocopied these effects, implicating one-carbon metabolism in the elevation of formaldehyde, although the proximal enzymatic source of formaldehyde was not identified. Together, these findings establish that oncogenic kinase signaling drives elevated endogenous formaldehyde production in transformed cells.

### 3.3. AML Depend on Detoxification and DNA Repair to Overcome Aldehyde-Genotoxicity

The dependence of AML cells on aldehyde detoxification was uncovered through the ex vivo transformation of primary murine HSPCs [115]. Lineage^−^ cKit^+^ HSPCs transformed with oncogenic tyrosine kinases (FLT3-ITD or JAK2-V617F) proliferated and formed colonies robustly when derived from wild-type mice, but exhibited 50–70% growth and colony formation defects when derived from *Aldh2*^−/−^ or *Adh5*^−/−^ mice [115]. This defect emerged specifically following leukemic transformation as *Aldh2*^−/−^ or *Adh5*^−/−^ mice do not exhibit gross HSPC defects at steady state [23,57,62,64,105]. The oncogenic kinase-transformed HSPCs also exhibited a dependency on polymerase theta (Polθ)-mediated end joining (TMEJ) [114,115], which resolves formaldehyde-induced DPCs and downstream DNA double-strand breaks [197]. The pharmacological or genetic inhibition of Polθ selectively suppressed the growth of oncogenic kinase-transformed HSPCs while sparing untransformed HSPCs [114]. Furthermore, combined Polθ loss with ALDH2 or ADH5 deficiency was synergistic. Oncogenic kinase-transformed HSPCs from *Aldh2*^−/−^ *Polq*^−/−^ and *Adh5*^−/−^ *Polq*^−/−^ double-knockout mice exhibited stronger growth suppression than either single knockout [115]. The same synergy was observed in human AML cells treated with combinations of Polθ inhibitors and ALDH2 or ADH5 inhibitors [115]. Critically, *Aldh2*^−/−^ *Polq*^−/−^ and *Adh5*^−/−^ *Polq*^−/−^ mice exhibited minimal hematopoietic defects despite the combined genetic loss, with no significant changes in peripheral blood counts, HSPC frequency, baseline γH2AX, or serial clonogenic activity compared to single knockouts. This indicates that normal HSPCs can tolerate combined aldehyde detoxification and Polθ deficiency in a way that oncogenic kinase-transformed HSPCs cannot. Together, these findings reveal that oncogenic kinase signaling rewires AML cells into a state of chronic formaldehyde production and consequent dependence on ALDH2/ADH5 and Polθ for survival. This transformation-induced metabolic dependence may apply to AML subtypes defined by constitutive kinase signaling, including FLT3-ITD-, JAK2-V617F-, and BCR-ABL-driven leukemias. The absence of this dependency in normal hematopoiesis underlies the therapeutic combinations explored later in this review.

### 3.4. Loss of ALDH2 Creates Dependency on the FA Pathway in AML

Given the dependency on aldehyde detoxification in AMLs, an unexpected finding was that a subset of AMLs downregulates the expression of *ALDH2* through recurrent epigenetic silencing [198]. This was revealed by a CRISPR screen across a panel of human AML cell lines, where FA genes were found to be essential for survival in AML cell lines lacking *ALDH2* expression. This finding effectively recapitulates the genetic interaction discovered in *Aldh2*^−/−^*Fancd2*^−/−^ mice, but in established human AML cells where ALDH2 loss is acquired epigenetically rather than genetically. The clinical relevance of this finding was supported by TCGA AML specimens, where *ALDH2* expression varied approximately 100-fold between the highest and lowest expressing 10% of cases, indicating that profound *ALDH2* silencing is a feature of human AML. Crucially, the same study showed that *ALDH2* silencing in AML is driven by the dense DNA hypermethylation of the *ALDH2* promoter in genomic regions that are hypomethylated in normal hematopoietic stem and progenitor cells [198]. Treatment with the DNA methyltransferase inhibitor 5-azacytidine restored *ALDH2* expression and rescued AML cells from FA pathway inactivation, providing causal evidence that the methylation drives the dependency. To confirm that this rescue was specifically due to *ALDH2* rather than other genes affected by demethylation, the ectopic re-expression of *ALDH2* in AML cell lines also rescued their FA dependency. Interestingly, the overexpression of other ALDH enzymes (*ALDH1A1*, *ALDH1A2*, *ALDH1A3*, *ALDH1B1*, *ALDH6A1*, and *ALDH7A1*), including a mitochondrially targeted *ALDH1A1* variant designed to match ALDH2’s compartmental localization, all failed to rescue. This suggests that the relevant genotoxic aldehyde is a specific substrate of ALDH2 not redundantly cleared by other ALDH paralogs. The re-expression of *ALDH2* reduced cellular MDA, while *ALDH1A1* expression did not, identifying endogenous MDA as a candidate genotoxic driver in AML, though further studies will be needed to confirm this. Overall, this study provides causal evidence that the loss of an ALDH enzyme in AML directly impacts genome stability and creates an acquired dependence on DNA repair. The finding offers much-needed proof of principle that the widespread dysregulation of aldehyde detoxification enzymes documented across other human cancers (Figure 1 and Figure 2) could have similar consequences for genome stability.

### 3.5. FA-Deficient Cancers Tolerate Rather than Mitigate Aldehyde Genotoxicity

The inactivation of DNA repair is a frequent and often early prerequisite event during the development of cancer. In many studies using cancer cell lines, the genetic inactivation of DNA repair results in hypersensitivity to aldehydes, indicating that aldehydes remain a genotoxic threat for cancer cells as well as non-malignant somatic cells [9,21,112,198,199]. This cell line sensitivity translates to selective aldehyde toxicity in vivo: acetaldehyde inhibited the growth of murine BRCA1/2-deficient breast tumor allografts and *BRCA2*-deleted human colorectal tumor xenografts, while their BRCA1/2-proficient counterparts were not significantly affected [200]. How do cancer cells harboring a loss of DNA crosslink repair pathways survive against endogenous aldehyde genotoxicity? One possibility is to relieve the endogenous aldehyde burden by reducing production or enhancing detoxification enzymes. Although elevated ALDH expression is observed in some human cancers (Figure 1), the evidence from cancers like EAC and AML where endogenous aldehydes are elevated [114,136] and aldehyde detoxification enzymes are downregulated [136,198] (Figure 1) does not support this model. An alternative possibility is that cancers tolerate aldehyde genotoxicity by suppressing the DNA damage response that would otherwise eliminate the cancer cell. Insight into this question comes from studies of FA-derived cancers, which develop in tissue constitutively deficient in DNA repair, thus providing a window into how cancer cells navigate elevated aldehyde genotoxicity.

FA patients develop a stereotyped spectrum of malignancies, most prominently AML and squamous cell carcinomas of the head and neck, esophagus, and anogenital region, at substantially elevated rates and younger ages than the general population [201,202]. The whole-genome and whole-exome sequencing of FA-derived squamous cell carcinomas established that the primary genomic signature of FA repair deficiency is a high burden of structural variants that drive somatic copy number alterations, with fewer single-nucleotide mutations than sporadic squamous cell carcinomas [203]. *TP53* was the most frequently mutated gene, found in over 80% of FA-derived HNSCCs [203,204]. Among the recurrent somatic copy number alterations, 38% of FA-derived HNSCCs harbored focal deletions of ALDH1B1 compared to only 5% of 415 human papillomavirus (HPV)-negative sporadic TCGA-HNSCCs [203], implicating selection rather than passenger consequence. In support of this, a differential gene expression analysis of FA-derived HNSCC versus sporadic HNSCCs revealed downregulation of multiple aldehyde detoxification enzymes, including *ALDH1A1*, *ALDH2*, *ALDH3A1*, and *ALDH3B2*, alongside an upregulation of DNA damage signaling (*ATM*, *ATR*, *CHEK1*), homologous recombination (*BRCA1*, *BRCA2*, *RAD51B*, *RAD54B*, *MRE11*), TMEJ (*POLQ*), and translesion synthesis (*REV3L*, *POLN*, *POLK*) [203]. This expression signature is consistent with FA-deficient cancers further losing aldehyde detoxification while upregulating residual DNA repair and lesion-tolerance pathways, suggesting selection for tolerance rather than enhancing aldehyde detoxification enzymes.

The structural variant mutational pattern in FA-derived HNSCCs [203] was recapitulated from a sequencing analysis of 335 FA patients with 62 cases of clonal evolution where FA-derived AML exhibited an elevated burden of somatic copy number alterations (CNA) compared to sporadic AML [205]. Furthermore, the CNA burden progressively increased along the clinical trajectory from bone marrow failure to myelodysplastic syndrome to overt AML. The most recurrent CNAs were chromosome 1q gain (52% of patients), which results in the trisomy of *MDM4*, a negative regulator of p53. The additional copy of *MDM4* provides a survival advantage for the pre-leukemic or leukemic clones emerging from an FA hematopoiesis landscape defined by p53 hyperactivation that depletes HSPCs and causes bone marrow failure [57,64,206]. Consistent with this, in *Fancg*^−/−^ mice carrying an extra allele of *MDM4*, HSPCs exhibited greater fitness, resistance to inflammation-mediated bone marrow failure, and clonal dominance when transplanted in competition with wild-type FA-like cells [205]. While the corresponding RNA-seq analysis confirmed the gene-dosage-dependent upregulation of *MDM4* and downmodulation of p53 target genes in 1q+ FA samples, aldehyde detoxification gene expression was not specifically examined, leaving open whether the systematic ALDH downregulation observed in FA-HNSCC extends to FA-AML.

Altogether, these data suggest that, within FA-deficient tissue, malignant cells are not selected for enhanced aldehyde detoxification to relieve aldehyde genotoxicity. Instead, the loss of aldehyde detoxification appears to be positively selected, through the deletion (*ALDH1B1* in FA-HNSCC) or systematic transcriptional downregulation of multiple ALDH enzymes, which would predict a further amplification of aldehyde genotoxicity to fuel cancer evolution. Critically, this is combined with loss of p53 pathway function (through *TP53* mutation in FA-HNSCC or *MDM4* amplification as a functional surrogate in FA-AML) to confer tolerance and persistence of cancer cells experiencing elevated aldehyde-driven genome instability. This conclusion is reinforced in *Aldh2*^−/−^ *Fancd2*^−/−^*Trp53*^−/−^ mouse model discussed earlier, in which genetic p53 loss rescues HSC depletion in the 2-tier-deficient background while accelerating leukemia development, providing a mouse-genetic analog to the *TP53*/*MDM4* alterations observed in FA patient tumors.

### 3.6. Exploiting Aldehyde Genotoxicity for Therapy in Cancer

Can we translate endogenous aldehyde genotoxicity to target cancer? The therapeutic exploitation of aldehyde genotoxicity has in fact been a feature of cancer chemotherapy for decades. Cyclophosphamide and ifosfamide, long-established chemotherapy agents, are prodrugs that generate the aldehyde intermediate aldophosphamide, which decomposes to release the alkylating agent phosphoramide mustard and the cytotoxic byproduct acrolein [207]. Chemoresistance to these agents frequently correlates with elevated ALDH1A1 expression that inactivates aldophosphamide [208,209,210]. Anthracyclines (doxorubicin, daunorubicin) similarly generate formaldehyde that contributes to their formation of cytotoxic DNA crosslinks [211,212]. These established chemotherapies validate the principle that elevating aldehyde burden in cancer cells can be therapeutic. Below, we review the mechanistic frameworks emerging from the study of aldehydes in cancer that present novel therapeutic opportunities to harness endogenous aldehydes for cancer therapy.

#### 3.6.1. Targeting 2-Tier Pathways to Elevate Aldehyde Genotoxicity

Pharmacological ALDH or ADH inhibition elevates cellular aldehyde levels, exposing cancer cells with compromised DNA repair to genotoxic stress. Several studies demonstrated a proof-of-concept use of inhibitors against ALDH or ADH5 with DNA repair inhibition in pre-clinical models with promising results. Disulfiram, an ALDH2 inhibitor [213], inhibited the growth of patient-derived xenograft cells from BRCA1-deficient breast cancers [200], demonstrating rationale for the selective targeting of aldehyde detoxification in tumors with pre-existing loss of tier 2 DNA repair. The same framework can be exploited by targeting both aldehyde detoxification and DNA repair for cancers with elevated endogenous DNA damage [115]. Significant anti-leukemic effects and prolonged survival were observed in leukemia-xenografted mice that received ADH5 inhibitor N6022 [214] or disulfiram combined with Polθ inhibitor RP-6685 [215]. Another potential 2-tier vulnerability could be offered by poly(ADP-ribose) polymerase (PARP) inhibitors, such as olaparib, which are licensed for BRCA-mutated and homologous-recombination-deficient cancers. Studies suggest PARP1 participates in the cellular response to aldehyde-induced DNA damage independently of FA/BRCA status. In FA/BRCA intact cells, the loss of PARP1 induces hypersensitivity to formaldehyde [199,216]. Thus, beyond their established use in homologous-recombination-deficient cancers, PARP inhibitor in combination with the inhibition of aldehyde detoxification enzymes represents a rational, as-yet-untested therapeutic strategy to harness genotoxic aldehydes. In addition, ALDH inhibition could combine with DDR inhibitors. Pharmacological ALDH1 inhibition with the pan-ALDH1A inhibitor 673A [170,217] combined with ATM/ATR inhibitors in ovarian carcinoma xenograft resulted in a significant reduction in tumor volume and increased γH2AX markers [170].

#### 3.6.2. Targeting Tolerance of Aldehyde Genotoxicity in Cancer

Cancers tolerating elevated aldehyde genotoxicity through suppressed DDR represent an additional therapeutic axis beyond directly targeting 2-tier protection pathways. The *MDM4* amplification driving FA-AML clonal evolution suppresses the p53 response that would otherwise eliminate aldehyde-damaged HSPCs. This was therapeutically harnessed through pharmacological p53 restoration with the MDM4/MDM2 inhibitor ALRN-6924, a stapled α-helical peptide that has previously been shown to activate the p53 pathway in AML [218,219]. The treatment of 1q+ FA-AML cells in culture with ALRN-6924 impaired growth and prolonged the S phase of the cell cycle, consistent with cell cycle arrest from p53 activation [205]. In vivo, the ALRN-6924 treatment of 1q+ FA-AML cells xenografted into immunodeficient mice resulted in a significant suppression of leukemic cell growth [205]. While this treatment holds promise for FA patients with 1q+ AML, the therapeutic window for leukemic cells may be narrow, as non-malignant FA HSPCs also exhibit an activation of p53, which could be detrimental if further activated by MDM4 inhibition [64,206].

## 4. An Updated 2-Tier Model of Aldehyde-Mediated Genome Protection

**Figure 3:**
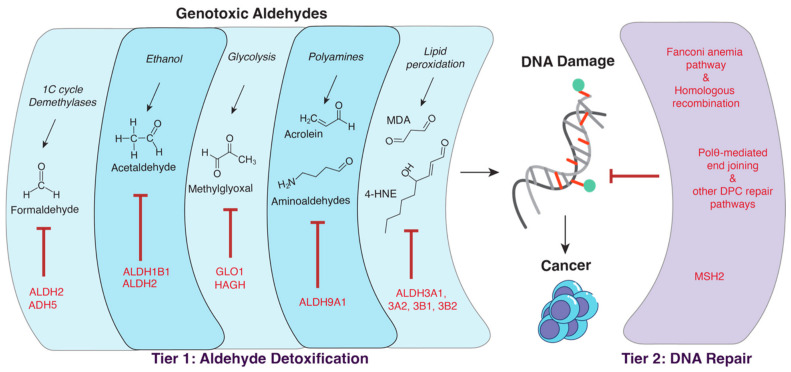
Updated 2-tier model of aldehyde-mediated genome protection. Genotoxic aldehydes arise from diverse metabolic sources: formaldehyde from the one-carbon cycle and demethylase reactions; acetaldehyde from ethanol metabolism; methylglyoxal from glycolysis; aminoaldehydes and acrolein from polyamine catabolism; and malondialdehyde (MDA) and 4-hydroxynonenal (4-HNE) from lipid peroxidation. In tier 1 (aldehyde detoxification), specific enzymes clear each aldehyde class (denoted by red blunt-ended arrows) before it can damage DNA: ALDH2 and ADH5 (formaldehyde), ALDH1B1 and ALDH2 (acetaldehyde), GLO1 and HAGH (methylglyoxal), ALDH9A1 (aminoaldehydes and acrolein), and ALDH3A1, 3A2, 3B1, and 3B2 (lipid peroxidation-derived aldehydes). When tier 1 detoxification is insufficient, aldehydes react with DNA to generate lesions, including interstrand crosslinks and DNA–protein crosslinks. In tier 2 (DNA repair), multiple pathways resolve these lesions: the Fanconi anemia pathway and homologous recombination; Polθ-mediated end joining (TMEJ) and other DNA–protein crosslink repair pathways; and MSH2-dependent repair. Failure of both tiers results in unresolved DNA damage, driving cancer development in non-malignant cells and fueling cancer evolution in malignant cells.

When originally proposed, the 2-tier model identified two complementary modes of defense against aldehyde genotoxicity: tier 1 involves enzymatic detoxification (ALDH2 and ADH5 clearing formaldehyde and acetaldehyde) and tier 2 involves DNA repair (the FA pathway resolving aldehyde-induced DNA crosslinks) [7,220]. While the loss of either aldehyde detoxification or DNA repair can be fully or partially compensated by the other respective tier of aldehyde defense, the combined loss of both tiers results in the amplification of aldehyde genotoxicity and cancer. This model was built on studies in hematopoietic stem cells and explained the aldehyde-induced bone marrow failure and leukemia predisposition. The evidence reviewed here supports a substantial expansion of this model, with additional players in both tiers exhibiting tissue specificity (Figure 3). First, several additional aldehyde detoxification enzymes now fulfill a tier 1 role in limiting additional endogenous genotoxic aldehydes beyond formaldehyde and acetaldehyde. ALDH1B1 demonstrates tier 1 defense against ethanol-derived acetaldehyde in the gastrointestinal tract to protect against intestinal tumors [91]. The increased ovarian tumor phenotype in *Aldh9a1*^−/−^ *Fanca*^−/−^ mice identifies ALDH9A1 as a tier 1 enzyme protecting against polyamine catabolism-derived aldehydes and acrolein [112]. In keratinocytes, the precursor cells of squamous cell carcinomas that afflict FA patients, ALDH3 family enzymes cooperate with the FA pathway to protect against DNA damage caused by lipid peroxidation-derived aldehydes such as 4-hydroxynonenal [147]. GLO1 is a non-ALDH enzyme that fulfills tier 1 defense against methylglyoxal-induced genotoxicity in breast epithelial cells [22].

Furthermore, the study of genetic interactions between aldehyde detoxification enzymes and DNA repair pathways has expanded the range of repair mechanisms encompassed by tier 2 beyond the Fanconi anemia pathway. Acetaldehyde-induced intestinal genotoxicity in *Aldh1b1*^−/−^ mice cooperates with mismatch repair to suppress intestinal tumorigenesis [91]. Polθ-mediated end joining (TMEJ) resolves the DPCs and associated double-strand breaks generated by elevated formaldehyde in oncogenic kinase-driven leukemia [114,115]. The methylglyoxal-induced genotoxicity in breast epithelium described above is contained by BRCA2-dependent homologous recombination, which acts downstream from FA-mediated repair aldehyde-DNA lesions. Interestingly here, the loss of tier 1 methylglyoxal clearance directly compromises the integrity of tier 2 through the methylglyoxal-mediated degradation of BRCA2 protein, revealing additional nodes of interaction within the 2-tier model [22]. While we have only included the DNA repair pathways that have demonstrated direct genetic interaction with aldehyde detoxification enzymes, additional repair pathways have been identified to protect against aldehyde genotoxicity, as discussed in detail in other reviews [13,14]. Therefore, we anticipate a further expansion of tier 2 as future studies dissect the interaction between these DNA repair pathways and aldehyde detoxification to protect against cancer.

## 5. Future Questions and Research Strategies

The studies synthesized here support a central role for aldehyde detoxification enzymes in limiting genome instability and restricting cancer formation. Three open questions deserve focused attention.

What are the endogenous aldehydes driving genome instability and cancer? ALDH enzymes act on a broad range of substrates, and exogenous aldehydes used to challenge ALDH-deficient experimental models may not be the same aldehydes driving endogenous genotoxicity in vivo. We have found the detection of endogenous aldehyde-DNA adducts to be a useful approach, since many physiological aldehydes form unique adducts of known mass and structure amenable to sensitive quantification by LC–MS. This strategy successfully identified endogenous formaldehyde as the major genotoxic aldehyde when both ADH5 and ALDH2 are lost [23]. Adduct elevation can also provide evidence for direct genotoxicity if the adduct is itself mutagenic; even for less mutagenic adducts like *N*^2^-hydroxymethyl-dG, their elevation indicates increased formaldehyde in the nuclear compartment. Unbiased DNA adductomic LC–MS approaches capable of quantifying hundreds of distinct adduct species could systematically reveal perturbations in the endogenous aldehyde landscape [221,222,223].

How does loss of aldehyde detoxification reshape the mutational landscape? Aldehyde-specific mutational signatures would both identify the relevant genotoxic aldehydes in detoxification-deficient cells and provide compelling evidence for aldehyde contributions to mutagenesis in cancer initiation and progression. At present, many cancer mutational signatures lack known etiology, and many characterized aldehyde-DNA adducts have not been linked to specific signatures. While LC–MS provides ultrasensitive adduct quantification, the requirement for hydrolysis to single nucleosides erases sequence context. Nanopore sequencing offers a promising alternative, where the direct sequencing of native DNA strands preserves both nucleobase identity and conjugated modifications [224]. This method already maps 5-methyldeoxycytosine and *N*^6^-methyldeoxyadenosine; expanding it to aldehyde-DNA adducts could reveal their enrichment at mutational hotspots and clarify how specific adducts are converted to mutations [225].

Which cancers harbor exploitable aldehyde-genotoxic vulnerabilities? Our pan-cancer analysis documents systematic dysregulation of aldehyde detoxification enzyme expression across diverse cancer types (Figure 2 and Figure 3), yet only a few cancers (EAC [136], AML [114,115,198], FA-derived cancers [203,205], BRCA-mutant cancers [22,200]) have been mechanistically examined for endogenous aldehyde-driven genome instability. Identifying additional vulnerable cancers requires integrated approaches: LC–MS adductomics of primary tumor samples to survey the endogenous aldehyde landscape, correlation of detoxification enzyme dysregulation with substrate accumulation, and functional testing of synthetic lethality with DNA repair inhibitors. The translational rationale is strong: endogenous aldehydes generated as obligate byproducts of cancer metabolic rewiring may be particularly difficult for cancer cells to bypass, making them attractive targets for the therapeutic strategies discussed above.

## 6. Conclusions

The recognition that reactive aldehydes are not merely passive metabolic by-products but active endogenous genotoxins, and that the enzymes clearing them constitute a first line of genome defense, represents a fundamental shift in how we understand the origins of cancer-associated genome instability. The ALDH, ADH, and glyoxalase families are not peripheral players in cancer biology; they are gatekeepers of genome integrity, and their disruption may be a major endogenous contributor to carcinogenesis. The expanded 2-tier model presented here extends a framework originally built on hematopoietic stem cells to a tissue-specific landscape in which multiple tier 1 enzymes, ALDH1B1 in intestine, the ALDH3 family in keratinocytes, ALDH9A1 in ovarian tissue, and GLO1 in breast epithelium, cooperate with diverse tier 2 repair mechanisms beyond the FA pathway. In established cancers, this protective architecture is dysregulated rather than restored: FA-derived cancers further lose aldehyde detoxification capacity while suppressing the p53 response that would otherwise eliminate them, and oncogenic kinase-driven AML cells become dependent on integrated detoxification and repair to survive their own elevated formaldehyde production. These dependencies define a therapeutic window in which the cancer cell’s own aldehyde burden can be turned against it.

## Data Availability

No new data were created or analyzed in this study.

## Abbreviations

ADH5	alcohol dehydrogenase 5 (also known as GSNOR)
AGT	O6-alkylguanine-DNA alkyltransferase (also known as MGMT)
ALDH	aldehyde dehydrogenase
AMeD	aplastic anemia, mental retardation and dwarfism syndrome
AML	acute myeloid leukemia
APE1	apurinic/apyrimidinic endonuclease 1
ATM	ataxia telangiectasia mutated
ATP13A3	adenosine triphosphatase 13A3
ATR	ataxia telangiectasia and Rad3 related
53BP1	p53 binding protein 1
BRCA	breast cancer gene
CHK1	checkpoint kinase 1
CNA	copy number alteration
CRISPR	clustered regularly interspaced short palindromic repeats
CSB	Cockayne syndrome B protein
DDR	DNA damage response
DNA-PKcs	DNA-dependent protein kinase catalytic subunit
DPC	DNA-protein crosslink
EAC	esophageal adenocarcinoma
EGA	European Genome–Phenome Archive
ESD	S-formylglutathione hydrolase
EXO1	exonuclease 1
FA	Fanconi anemia
FALDH	fatty aldehyde dehydrogenase (ALDH3A2)
FANC	Fanconi anemia complementation group
FISH	fluorescence in situ hybridization
GABA	γ-aminobutyric acid
GEO	Gene Expression Omnibus
GLO1	glyoxalase 1
GLO2	glyoxalase 2 (also known as HAGH)
GSH	glutathione
GSNO	S-nitrosoglutathione
GSNOR	S-nitrosoglutathione reductase (also known as ADH5)
GTEx	Genotype-Tissue Expression
HAGH	hydroxyacylglutathione hydrolase (also known as GLO2)
γH2AX	phosphorylated H2A histone family member X
HNSCC	head and neck squamous cell carcinoma
4-HNE	4-hydroxynonenal
HPV	human papillomavirus
HR	homologous recombination
HSC	hematopoietic stem cell
HSPC	hematopoietic stem and progenitor cell
IARC	International Agency for Research on Cancer
ICL	interstrand crosslink
iNOS	inducible nitric oxide synthase
LC–MS	liquid chromatography-mass spectrometry
MDA	malondialdehyde
MG	methylglyoxal
MGMT	O6-methylguanine-DNA methyltransferase (also known as AGT)
MMR	mismatch repair
MLH1	MutL homolog 1
MSH	MutS homolog
MutLα	MutL homolog complex (MLH1–PMS2)
MutSα	MutS homolog complex (MSH2–MSH6)
NAD^+^	nicotinamide adenine dinucleotide
NER	nucleotide excision repair
OGG1	8-oxoguanine DNA glycosylase 1
PARP	poly(ADP-ribose) polymerase
PMS2	postmeiotic segregation increased 2
Polθ	DNA polymerase theta
ROS	reactive oxygen species
SCC	squamous cell carcinoma
SHMT1/2	serine hydroxymethyltransferase 1/2
SLS	Sjögren–Larsson syndrome
SOD	superoxide dismutase
TARGET	Therapeutically Applicable Research to Generate Effective Treatments
TCA	tricarboxylic acid
TCGA	The Cancer Genome Atlas
TLS	translesion synthesis
TMEJ	Polθ-mediated end joining
WT	wildtype
XRCC1	X-ray repair cross-complementing 1
